# Susceptibility to short-term ozone exposure and cardiovascular and respiratory mortality by previous hospitalizations

**DOI:** 10.1186/s12940-018-0384-z

**Published:** 2018-04-13

**Authors:** Auriba Raza, Marcus Dahlquist, Tomas Lind, Petter L. S. Ljungman

**Affiliations:** 1 Institute of Environmental Medicine, Karolinska Institutet, Nobels väg 13 | Box 210 |, SE-171 77 Stockholm, Sweden; 2 Center for Occupational and Environmental Medicine, Stockholm County Council, Solnavägen 4, 113 65 Stockholm, Sweden; 3 Department of Cardiology, Danderyd Hospital, Stockholm, Sweden

**Keywords:** Air pollution, Cardiovascular deaths, Respiratory deaths, Susceptibility

## Abstract

**Background:**

Ozone (O_3_) has been associated with cardiorespiratory mortality although few studies have explored susceptible populations based on prior disease.

We aimed to investigate the role of previous hospitalization on the association between short-term exposure to O_3_ and cardiovascular (CV) and respiratory mortality.

**Methods:**

We performed time series analyses using generalized additive models and case-crossover on 136,624 CV and 23,281 respiratory deaths in Stockholm County (1990–2010). Deaths were linked to hospital admissions data. We constructed 2-day and 7-day averages using daily 8-h maximum for O_3_ and hourly values for PM_2.5_, PM_10_, NO_2_, and NO_x_ from a fixed monitor.

**Results:**

We observed a 0.7% (95% CI: 0.1%, 1.3%) and 2.7% (95% CI: 0.8%, 4.6%) higher risk of CV and respiratory death per 10 μg/m^3^ higher 2-day and 7-day average O_3_ respectively. Individuals previously hospitalized for myocardial infarction demonstrated 1.8% (95% CI: 0.4%, 3.4%) higher risk of CV death per 10 μg/m^3^ higher 2-day average O_3_ and similar associations were observed in individuals with no previous hospitalization for any cause. Individuals with previous hospitalizations did not show susceptibility towards O_3_-related risk of respiratory mortality. We observed no associations for other pollutants.

**Conclusion:**

Short-term ozone exposure is associated with CV and respiratory mortality and our results may suggest higher susceptibility to CV mortality following O_3_ exposure in individuals previously hospitalized for myocardial infarction. Higher risks were also observed in individuals with cardiovascular death as their first presentation of disease.

**Electronic supplementary material:**

The online version of this article (10.1186/s12940-018-0384-z) contains supplementary material, which is available to authorized users.

## Background

Higher levels of ambient Ozone (O_3_) have been associated with cardiovascular (CV) and respiratory mortality [1, 2]. Severity of health effects of O_3_ experienced by individuals with pre-existing diseases or other determinants may be larger than among other individuals [3]. Knowledge on susceptible populations is important for providing relevant guidelines for air quality standards protecting significant susceptible groups within the general population. In an attempt to identify such groups, some studies have explored age differences and reported elderly [3–6] and children [7] as populations with higher risk. Susceptibility according to sex or other sociodemographic characteristics [3, 4, 8] have also been explored but with mixed results. Previous studies that have explored the impact of pre-existing diseases on the susceptibility to O_3_ mortality have for example used surrogates for pre-existing diseases using secondary diagnosis of death [3] or have considered total mortality rather than cause-specific mortality, both factors affecting accurate identification of susceptible populations [3, 6, 8, 9]. The pre-existing diseases that conferred increased susceptibility in these studies were not fully consistent from study to study but centered around diabetes, atrial fibrillation and atherosclerotic diseases. In addition, there is some evidence to suggest that susceptibility factors to O_3_ mortality are more important in cities with low air pollution concentrations [3].

Illness related to CV or respiratory diseases resulting in hospitalizations such as ischemic heart disease events, exacerbations of chronic obstructive lung disease (COPD), and diabetes are likely to be harbingers of CV or respiratory mortality and may be markers of susceptibility to the effects of O_3_. In a previous study from Stockholm, we observed association between 2-day levels of O_3_ and risk of non-traumatic mortality and this risk was higher among individuals with previous hospitalizations for acute myocardial infarction (AMI) [8]. We therefore hypothesized that individuals with previous hospitalizations for AMI, other CV diseases, respiratory diseases, and diabetes would carry higher risks of CV mortality per incremental increase in short-term O_3_ exposure compared to the risk in the overall population. Likewise, we hypothesized that individuals with previous hospitalizations for COPD, pneumonia, or other respiratory diseases would carry higher risks of respiratory mortality per incremental increase of short-term O_3_ exposure compared to the risk in the overall population. Consequently, in the present study we aimed to investigate the role of previous hospitalizations on the associations between short-term exposure to O_3_ and CV and respiratory mortality. In the setting of the low-level pollution environment of Stockholm, Sweden, we made use of the well-validated Swedish death registry and inpatient registry [10] to include all deaths in Stockholm and all hospitalizations in Sweden to study these associations using continuous monitoring of ambient air pollution concentrations.

## Methods

### Study population

We included residents of Stockholm County older than 30 years with CV or respiratory deaths occurring in the County from 1990 to 2010. We obtained age, sex, date and cause of death, and all dates and diagnoses of hospitalizations from the Swedish Board of Health and Welfare with mortality and hospitalizations data available from 1990 and 1987, respectively. We linked all CV and respiratory deaths to all previous hospitalizations in Sweden up to 3 years before death to create susceptible groups based on the principal diagnosis code (International Classification of Diseases, Ninth Revision (ICD-9) and Tenth Revision (ICD-10) at discharge (Table 1). In a previous study, hospitalizations prior to 3 years before death did not identify a population sensitive to air pollution related overall mortality [8] and therefore we chose a 3 year period for previous hospitalizations in order to have a uniform hospitalization period for all individuals.

**Table 1:** Characteristics and categorization of cardiovascular (ICD-9 390–459; ICD-10 I00-I99) and respiratory (ICD-9 460–519; ICD-10 J00-J99) deaths by previous admissions for diseases among Stockholm County residents ≥30 years old, 1990–2010

Categorization of mortality	Diagnosis of hospital admission		Total	Percent	Mean age (SD)	Female (%)
	ICD-9	ICD-10				
A. Total cardiovascular deaths			136,624	100	81 (10)	54.1
Individuals previously hospitalized for						
i) AMI	410	I21-I22	20,948	15.0	81 (11)	47.3
ii) Non-AMI CVD	390–409, 411–459	I00-I20, I23-I99	60,346	44.2	81 (10)	54.7
iii) Respiratory diseases	460–519	J00-J99	5919	4.4	81 (10)	51.2
iv) Diabetes	250	E10-E14	1081	1.0	81 (10)	51.4
v) Other diseases	All except above	All except above	24,283	18.0	81 (10)	60.1
vi) Not hospitalized in 0–3 years^a^			16,190	12.0	83 (11)	58.0
vii) Not hospitalized since 1987			7417	5.4	73 (13)	41.2
B. Total respiratory deaths			23,281	100	80 (10)	53.1
Individuals previously hospitalized for						
i) COPD	490–496	J40-J47, J67	5774	25.0	80 (11)	52.6
ii) Pneumonia	480–486	J12-J18	6003	26.0	79 (11)	45.6
iii) Other respiratory diseases	460–480, 486–490, 497–519	J00-J11, J19-J40, J48-J66, J68-J99	1648	7.0	80 (11)	49.8
iv) Other diseases	All except above	All except above	6774	29.0	79 (11)	56.4
v) Not hospitalized in 0–3 years^a^			2442	10.0	84 (11)	65.8
vi) Not hospitalized since 1987			640	3.0	80 (14)	54.0

Note: All categories are mutually exclusive

^a^These individuals had hospitalizations between 1987 and up to 3 years preceding their death but not in the 3 years preceding death

### Categorization by previous hospitalization

For individuals who died a CV death, we defined susceptibility based on hospitalizations for i) acute myocardial infarction (AMI; ICD-9 410, ICD-10 I21-I22); ii) all other cardiovascular diseases (CVD; ICD-9 390–409, 411–459, ICD-10 I00–20, I23–99), excluding individuals who had AMI; iii) respiratory diseases (ICD-9 460–519, ICD-10 J00–99), excluding individuals with CVD; iv) diabetes (ICD-9 250, ICD-10 E10–14), excluding individuals with CVD and respiratory diseases; v) other diseases, excluding individuals who have been hospitalized for CVD, respiratory diseases, and diabetes; vi) individuals who were not hospitalized between 0 and 2 years before death for any disease but had earlier hospitalizations; and vii) individuals who died cardiovascular death but had not been hospitalized since 1987 for any cause.

For individuals who died a respiratory death, we defined susceptibility based on hospitalizations for i) chronic obstructive pulmonary disease (COPD; ICD-9 490–496, ICD-10 J40–47, J67); ii) pneumonia (ICD-9 480–486, ICD-10 J12-J18), excluding individuals who had hospitalizations for COPD; iii) other respiratory diseases (ICD-9 460–480, 486–490, 497–519, ICD-10 J00–11, J19–40, J48–66, J68–99), excluding individuals who had been hospitalized for COPD and pneumonia; iv) any disease other than respiratory disease; v) not hospitalized between 0 and 2 years but had earlier hospitalizations; and vi) individuals with respiratory deaths but no record of previous hospitalizations since 1987 for any cause.

In an effort to avoid double counting of individuals with multiple hospitalizations in different hospitalization categories we followed the above-mentioned hierarchies to ensure mutually exclusive groups. Only one hospitalization was considered for each individual and category. Count data was created per day for each mortality outcome and further stratified by categories of previous hospitalizations.

### Air pollution and meteorological data

Exposure data was provided by the Stockholm-Uppsala County Air Quality Management Association. We identified a priori O_3_ as our main exposure of interest based on our previous study and included exposure data for Nitrogen dioxide (NO_2_), Nitrogen oxides (NO_x_), particles with aerodynamic diameter smaller than 2.5 and 10 μm (PM_2.5_ and PM_10_) for comparison with other studies. Hourly means of O_3_, NO_2_, NO_x_, PM_2.5_, PM_10_, temperature and relative humidity were obtained from a single centrally located urban background monitoring station.

For O_3_, we first calculated an 8-h running mean over 24-h period to compute an 8-h maximum. We calculated 24-h averages of NO_2_, NO_x_, PM_2.5_, PM_10_, temperature, and relative humidity from hourly values.

### Statistical analysis

We performed both time series and case-crossover analyses to analyze associations between air pollutants and the risk of cause-specific mortality in those with and without previous hospitalizations. In time series, we employed generalized additive models based on quasi-Poisson distribution. These models were adjusted for long-term and seasonal trends, temperature, relative humidity, daily influenza hospital admissions, day of the week, and public holidays. We modeled seasonal and long-term time trend by penalized splines with 5 degrees of freedom (df) per year; holidays and day of week as indicator variables; the mean values of the relative humidity during the day of death and previous day as linear terms; the mean values of the temperature during the day of death and previous day as cubic splines with 3 degrees of freedom; and a 7-day running average of daily influenza admissions in Stockholm as cubic spline with 4 df.

For comparison we also employed the case-crossover analyses for both the main effects assessing associations between O_3_ exposure and CV or respiratory mortality and to estimate the presence of effect modification by season, age, sex, and susceptibility by previous hospitalizations using multiplicative terms including O_3_ and the specific effect modifier in separate models. We used a time stratified referent selection strategy with conditional logistic regression. Control periods were matched with case periods on same day of the week, within the same calendar month, and year as the day of death. This way, time trend and slowly changing individual characteristics were adjusted by the design. We adjusted for temperature and daily influenza hospital admissions as a restricted cubic spline with 2 degrees of freedom (df) and relative humidity as a linear term. Age was dichotomized by median age at the time of death and season by warm (April to September) and cold (October to March) season. For susceptibility by previous hospitalization, we performed a likelihood ratio test to compare the model with an interaction term including hospitalization categories (using the largest category as the reference) with the reduced model without the covariate containing the hospitalization categories.

We used a 0–1 day lag (2 day average) and 0–6 day lag (7-day average) in single-pollutant models in our initial analysis based on evidence from previous studies indicating most apparent effects both within the immediately preceding days and up to a week [5, 6]. We then selected a lag for subsequent analyses of CV and respiratory mortality that demonstrated the strongest association, in separate models, assessed by largest effect estimates with statistical significance. We also evaluated two-pollutant models to adjust for other pollutants. We then proceeded with stratified analyses of CV and respiratory mortality according to previous hospitalizations in time series analyses and separately employed the whole dataset in case-crossover analyses using a multiplicative term for O_3_ and hospitalization category.

In sensitivity analyses, we controlled for the effects of temperature over longer periods using averaging times of 0–6 days for temperature. Our cause-specific mortality data starts from 1990 and previous hospitalization data starts from 1987. We relaxed our criterion of a three-year uniform hospitalizations prior to death to include up to five-year previous hospitalizations for the purpose of comparing results to our previous study [8].

### Exploratory analyses

All exploratory analyses were performed post hoc in the subgroup of CV mortalities in individuals hospitalized for AMI. Since the timing of the hospitalization in relation to date of death may be important in modulating the susceptibility to short-term O_3_ exposure, we explored patterns of association by stratifying the time lapse between the last AMI hospitalization before death and death into four one-week categories and six six-month categories before death and employed models for each time-category. Each time-category included a separate model of daily CV deaths for individuals with AMI hospitalizations occurring within in specified time-period before date of death. For example, the first model included daily counts of CV deaths in a dataset in which CV deaths had been preceded with last AMI hospitalizations within 0 to 6 days (week 0) before death. In this manner, we created datasets for each stratum and performed stratified analysis.

All estimates are expressed as percent increase risk of death risk with 95% confidence intervals per 10 μg/m^3^ increase in air pollution levels. All case-crossover and time series analyses were performed using Stata and R, respectively.

## Results

A total of 159, 905 CV and respiratory deaths were observed in Stockholm County from 1990 to 2010. We observed 6 times more CV deaths compared to respiratory deaths with a slight majority occurring in women (Table 1). The average age at the time of CV death was 77 years in men and 84 years in women and at the time of respiratory death 79 years in men and 82 years in women.

Average concentrations of all measured pollutants were low by international comparison (Table 2). Ozone measurements showed a near-normal distribution with sizable temporal variability whereas other pollutants demonstrated right-skewed distributions. Ozone was moderately positively correlated with PM_10_, and negatively correlated with NO_2_ and NO_x_. Ozone and temperature were also moderately correlated (Table 3).

**Table 2:** Distribution of air pollution concentrations and meteorological parameters in 2-day (lag 0–1) and 7-day average (lag 0–6), Stockholm, Sweden, 1990–2010

Parameters	2-day average				7-day average			
	Mean (SD)	Min	Max	IQR	Mean (SD)	Min	Max	IQR
O_3_	62.8 (20)	4.7	143.0	27.7	62.7 (18)	11.7	121.9	27.0
PM_2.5_	8.2 (5)	0.9	41.6	4.6	8.2 (4)	2.2	39.8	3.9
PM_10_	15.3 (8)	2.2	92.7	8.8	15.3 (7)	3.9	64.2	7.4
NO_2_	19.0 (8)	2.4	78.7	11.3	19.1 (7)	4.6	56.9	9.3
NO_x_	26.9 (18)	2.5	404.4	18.3	27.1 (15)	5.1	239.4	16.3
Temp	7.6 (7)	−15.9	26.1	12.3	–	–	–	–
Rh	74.7 (12)	33.7	99.3	17.7	–	–	–	–

*O*_*3*_ ozone, *PM*_*2.5*_ mass concentration of particles ≤2.5 μm in aerodynamic diameter, *PM*_*10*_ mass concentration of particles ≤10 μm in aerodynamic diameter, *NO*_*x*_ nitrogen oxides, *NO*_*2*_ nitrogen dioxide, *Temp* temperature, *Rh* relative humidity

**Table 3:** Pearson correlation coefficients between air pollutants and meteorological parameters measured in Stockholm, Sweden, 1990–2010. Values above diagonal are for 7-day averages and below for 2-day averages

7-day	O_3_	PM_2.5_	PM_10_	NO_2_	NO_x_	Temp	Rh
2-day							
O_3_	1.00	0.26	0.47	−0.34	−0.43	0.50	−0.57
PM_2.5_	0.24	1.00	0.74	0.26	0.20	0.02	0.02
PM_10_	0.41	0.74	1.00	0.17	0.09	0.03	−0.23
NO_2_	− 0.32	0.23	0.23	1.00	0.90	− 0.37	0.09
NO_x_	−0.40	0.17	0.16	0.88	1.00	−0.34	0.11
Temp	0.47	0.04	0.04	−0.33	− 0.29	1.00	− 0.38
Rh	−0.60	0.04	−0.24	0.18	0.10	−0.38	1.00

*O*_*3*_ ozone, *PM*_*2.5*_ mass concentration of particles ≤2.5 μm in aerodynamic diameter, *PM*_*10*_ mass concentration of particles ≤10 μm in aerodynamic diameter, *NO*_*x*_ nitrogen oxides, *NO*_*2*_ nitrogen dioxide, *Temp* temperature, *Rh* relative humidity

We observed associations between 8-h maximum O_3_ levels and CV mortality for both averaging periods with narrower confidence intervals for 8-h maximum O_3_ averaged over 2 days before death and similar estimates in both in time series analysis and case-crossover analyses (Table 4). No associations were observed between average PM_2.5_, PM_10_, and NO_2_ levels and CV mortality regardless of averaging period or analyses method. In two-pollutant models, the association between O_3_ and CV mortality was slightly increased while estimates for PM_2.5_, NO_2_ and NO_x_ remained non-significant (Additional file 1: Table S1). In the two-pollutant model with PM_10_, we observed a slightly stronger estimate for O_3_ (0.9% increased risk per 10 μg/m^3^; 95% CI 0.2%, 1.6%) and unexpectedly a decreased risk of CV mortality related to PM_10_ (-1.3%; 95% CI: -2.4%, − 0.2%, per 10 μg/m^3^ increment).

**Table 4:** Percent change in cardiovascular and respiratory mortality in all subjects associated with a 10 μg/m^3^ increase in 2-day and 7-day average air pollution concentrations, respectively using time series and case-crossover design

Pollutant	Lags	Time series		Case-crossover	
		Cardiovascular mortality	Respiratory mortality	Cardiovascular mortality	Respiratory mortality
O_3_	0–1 day	0.7 (0.1, 1.3)	1.1 (−0.4, 2.6)	1.2 (0.6, 1.8)	1.2 (−0.2, 2.7)
	0–6 day	0.8 (0.1, 1.6)	2.7 (0.8, 4.5)	1.1 (0.3, 1.9)	2.5 (0.6, 4.4)
PM_2.5_	0–1 day	0.0 (−2.0, 2.1)	−0.1 (−4.7, 4.8)	− 0.2 (−2.2, 1.9)	−0.2 (−4,8, 4.7)
	0–6 day	− 0.3 (− 2.9, 2.4)	−4.1 (−9.9, 2.1)	0.4 (− 2.2, 3.0)	−8.1 (−13,6, − 2.3)
PM_10_	0–1 day	− 0.8 (−1.8, 0.2)	0.3 (− 2.1, 2.8)	− 1.0 (− 2.0, 0.0)	− 0.2 (− 2.7, 2.3)
	0–6 day	− 0.8 (− 2.1, 0.6)	−0.1 (−3.3, 3.2)	−0.5 (− 2.0, 0.9)	− 1.8 (−5.0, 1.5)
NO_2_	0–1 day	−0.6 (− 1.7, 0.5)	0.2 (− 2.4, 2.9)	−1.2 (− 2.3, 0.0)	0.2 (− 2,5, 3.0)
	0–6 day	0.3 (− 1.3, 1.9)	− 2,3 (−6.0, 1.5)	0.6 (− 1.1, 2.3)	−0.5 (− 4.4, 3.5)
NO_x_	0–1 day	− 0.1 (− 0.5, 0.4)	−0.1 (− 1.1, 0.9)	−0.4 (− 0.8, 0.1)	−0.1 (− 1,2, 1.1)
	0–6 day	0.1 (− 0.6, 0.7)	−1.3 (− 2.8, 0.3)	0.0 (− 0.6, 0.7)	−1.2 (− 2.8, 0.4)

*O*_*3*_ ozone, *PM*_*2.5*_ mass concentration of particles ≤2.5 μm in aerodynamic diameter, *PM*_*10*_ mass concentration of particles ≤10 μm in aerodynamic diameter, *NO*_*x*_ nitrogen oxides, *NO*_*2*_ nitrogen dioxide

In further analyses, 2-day average O_3_ was associated with a higher CV mortality risk in individuals older than the median age (82 years old, 1.7% (95% CI 0.9%, 2.4%) compared with individuals equal to or below the median age (0.6%; 95% CI -0.2%, 1.4%) with a *p*-value for interaction of 0.04 (Additional file 1: Table S2). Differences in O_3_ associations between men and women or deaths occurring in the warm or cold season were not as clear (*p*-values for interaction 0.7 and 0.9 respectively).

**Fig. 1:**
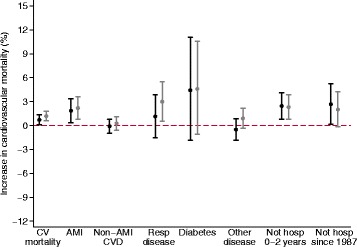
Percent change (95% confidence interval) in cardiovascular mortality associated with a 10 μg/m^3^ increase in 2-day average of 8-h maximum O_3_ concentrations by previous hospitalizations for acute myocardial infarction (AMI), other cardiovascular diseases excluding AMI (Non-AMI CVD), respiratory diseases, diabetes, other diseases, or by absence of any hospitalization between 0 to 3 years, or since 1987. All categories are mutually exclusive. Black markers indicate results from time series analyses and grey markers from case-crossover analyses

Associations between 2-day O_3_ and CV mortality by categories of hospitalization demonstrated positive associations in individuals with previous hospitalizations for AMI and in individuals who had not been hospitalized between 0 to 2 years before cardiovascular death or who had never been hospitalized since 1987 (Fig. 1). Previous hospitalization for respiratory disease seemed to confer greater risk for CV death following O_3_ exposure in the case-crossover analyses, however this was not corroborated in the time series analysis method. No associations were observed between O_3_ exposure and CV death in individuals with prior hospitalizations for CVD other than AMI, diabetes or any other disease. Risk increases per 10 μg/m^3^ O_3_ in AMI was roughly 2.5-fold higher estimate than for all CV deaths. For example, in individuals with previous hospitalizations for AMI, a 10 μg/m^3^ increment of 2-day average 8-h maximum O_3_ increased the risk of CV mortality by 1.8% (95% CI 0.3%, 3.0%) and 2.2% (95% CI 0.8%, 3.6%) for time series and case-crossover respectively. Comparing case-crossover models with and without interaction terms indicated likely interaction by hospitalization category (*p*-value for likelihood ratio test 0.02).

**Fig. 2:**
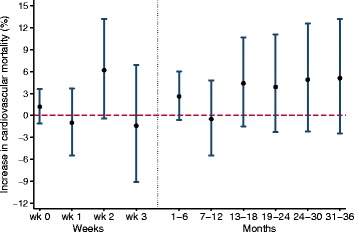
Percent change (95% confidence interval) in cardiovascular mortality associated with a 10 μg/m^3^ increase in 2-day average of 8-h maximum O_3_ concentrations in individuals with prior hospitalization for acute myocardial infarction. Individuals were stratified according to the time period between their last hospitalization and death. Strata included month 0 (0–28 days before death) divided into 4 separate weeks, and preceding months (1–36) grouped into 6-month periods. All strata are mutually exclusive and results are from time series analysis

We further stratified individuals with previous AMI hospitalization based on the admission date of the last AMI in time series analyses. The risk of CV mortality following 2-day average O_3_ did not demonstrate a clear trend for hospitalization occurring the month immediate preceding death. Risk estimates among individuals with hospitalizations occurring 1–36 months were consistently positive (Fig. 2).

We observed stronger associations between 8-h maximum O_3_ levels and respiratory mortality in 0–6 day lag compared to 0–1 day lag irrespective of analysis method (Table 4). We did not observe associations between average PM_2.5_, PM_10_, and NO_2_ levels and respiratory mortality for any averaging period. In two-pollutant models, associations between O_3_ exposure and respiratory mortality remained positive in the co-presence of NO_2_, and NO_x_ (Additional file 1: Table S1). In the two-pollutant model including PM_2.5_ and PM_10_, the association for O_3_ became weaker and non-significant and the estimates for both particulate matter fractions were reduced.

In further exploratory analyses associations between 7-day average O_3_ and respiratory mortality remained unchanged in models including a multiplicative term for O_3_ and sex, age, or season (*p*-values for interaction ≥0.1, Additional file 1: Table S3).

**Fig. 3:**
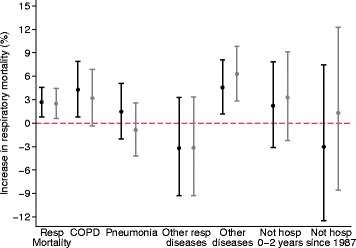
Percent change (95% confidence interval) in respiratory mortality associated with a 10 μg/m^3^ increase in 7-day average of 8-h maximum O_3_ concentrations by previous hospitalizations for chronic obstructive pulmonary disease (COPD), pneumonia, other respiratory diseases, other diseases, or by absence of any hospitalization between 0 to 3 years, or since 1987. All categories are mutually exclusive. Black markers indicate results from time series analyses and grey markers from case-crossover analyses

Associations between 7-day O_3_ exposure and respiratory mortality by previous hospitalization categories were similar in time series and case-crossover methods (Fig. 3). Positive associations were observed in individuals with previous hospitalization for COPD and other diseases, however; there was little support for effect modification by previous hospitalization (*p*-value for likelihood ratio test 0.63).

Associations were similar when modeling the same averaging periods for temperature as for exposure as well as the use of 5-year rather than 3-year previous hospitalizations data for vulnerable population subgroups.

## Discussion

Among all deaths in Stockholm County between 1990 and 2010 we observed associations between higher short-term concentrations of O_3_ and CV and respiratory mortality. Estimates were higher for respiratory than for CV mortality. Associations between O_3_ and CV mortality were relatively stronger in individuals hospitalized for AMI as well as for those individuals who had not been hospitalized for any disease. Associations between O_3_ and respiratory mortality were not affected by individuals’ previous hospitalizations. We did not observe associations between PM_2.5_, PM_10_, NO_2_ and NO_x_ levels and CV or respiratory mortality.

Associations for CV mortality were similar for both averaging periods of 8-h maximum O_3_. In contrast, we observed higher estimates for the association between 7-day average 8-h maximum O_3_ and respiratory mortality compared to the shorter averaging period of 2-day. This pattern is largely consistent with the The Air Pollution and Health: a European Approach study that also reported stronger estimates for the association between O_3_ and respiratory mortality in longer lags (0–20 day) as compared to shorter lags (0 and 0–1 day), while the opposite was observed for CV mortality [11]. This implies that exposure to high O_3_ levels has a more immediate effect on triggering CV mortality, whereas the effect on respiratory mortality might require longer O_3_ exposure and other biological processes [11].

Previous studies have reported positive associations between PM_2.5_, PM_10_, NO_2_ and NO_x_ and CV and respiratory mortality [12, 13]. We found no evidence of associations for these pollutants in our study. Possible explanations may include low concentrations and the use of single monitors to assess pollutants with more spatial heterogeneity than O_3_.

Little research has been conducted on identifying individuals susceptible to the harmful effects of air pollution based on previous CVD, however, our results are generally concordant with results from the existing studies. In our previous study from Stockholm [8], we observed higher total mortality in association with 8-h maximum O_3_ exposure in individuals with previous AMI compared to the full study population. Risk estimates are similar in the current study investigating CV mortality (1.7% vs 1.8%). A study from Italy [6], also investigating total mortality, reported non-significant associations between 0 and 5 day 8-h maximum O_3_ and total mortality in individuals with previous AMI hospitalization (2.6% increase risk per 10 μg/m^3^ 95% CIs: − 1.0%, 6.4%). However, diabetes was the pre-existing condition that conferred the greatest susceptibility to ozone, results which were not confidently confirmed in our analyses. Another study from Italy [9] investigating a more specific outcome, reported a borderline significant association between O_3_ exposure and out-of-hospital coronary deaths in individuals who had previous hospitalizations for ischemic heart disease (*p*-value 0.059) which lends some support to our finding that suggest susceptibility to CV mortality in individuals with previous hospitalization to AMI. In contrast to our results, a large multicity study from the US using secondary cause of death as a proxy for pre-existing disease reported no increased susceptibility between O_3_ and mortality for atherosclerotic conditions, however additional susceptibility was observed for atrial fibrillation [3].

Our results suggest that association between O_3_ exposure and CV death in individuals with AMI hospitalizations appeared to be driven mainly by AMI hospitalizations more than 1 month preceding death. Studies have reported the highest post AMI mortality within the first 28 days [14]. During this time patients are typically under intensive medication and the increased antithrombotic medication may potentially reduce the harmful effects of O_3_. We may also speculate that other risks are more dominant within the first month post-AMI compared to the effect of O_3_. In addition, individuals are plausibly less frequently outdoors during the first month after an AMI and they may be less exposed to outdoor O_3_ concentrations. Hence, the increased risk observed in individuals with AMIs more than 1 month preceding death may reflect an increased exposure to O_3_ or less exposure misclassification.

Out-of-hospital CV deaths with no previous hospitalization for any causes demonstrated an indication of association with O_3_ exposure. It is likely that many of these individuals had an undiagnosed (and untreated) underlying condition that made them susceptible to O_3_ exposure. Epidemiological studies as well as autopsy series reported presence of structural heart disease, including coronary artery disease, in 50–95% of adults who suffered sudden cardiac death without previously known heart disease [15, 16]. In our study, CV death in individuals not hospitalized since 1987 were relatively younger (mean age 73 years) compared to the rest of the individuals. Younger individuals are more likely to be exposed to higher O_3_ levels through outdoor physical activity. In addition, in a previous study in Stockholm based on a cardiac arrest registry, we reported associations between higher short-term exposure to O_3_ and risk of out-of-hospital cardiac arrest [17], largely supporting the theory that the previously non-hospitalized deaths may reflect cardiac arrests in individuals with latent heart disease.

Ozone exposure has been consistently associated with respiratory mortality in several studies including large multi-city studies [1, 4, 11, 18–20]. Associations have generally been reported from exposure periods between 0 and 1 and 0–5 day lags and our estimates of 2.7% higher risk per 10 μg/m^3^ 7-day exposure are in line with previously reported estimates. In addition to studies investigating O_3_ exposure and respiratory mortality, several studies have reported associations between O_3_ exposure and hospital admission for COPD [21–26].

Few previous studies have investigated the role of previous hospitalizations for respiratory disease on association between O_3_ and respiratory mortality. None of these have reported increased risk of death in individuals with previous respiratory hospitalizations [3, 6, 9]. In individuals with COPD hospitalizations, the timing of hospitalizations did not seem important in determining the susceptibility to O_3_ in relation to respiratory mortality.

We observed an increased risk of CV mortality among elderly following short-term exposure to O_3_. Other studies have also reported elderly as susceptible to the detrimental effects of O_3_ exposure [6, 8, 9, 27]. We did not observe risk difference in men or women, while, a majority of studies have reported stronger associations between O_3_ exposure and CV outcomes in women [3, 6], although some inconsistencies remain [8] and studies investigating specific pathophysiological mechanisms are lacking [3]. Furthermore, we observed no evidence to support differing risk to O_3_-related risk of respiratory mortality by age and sex.

Ozone has a strong seasonal trend with high levels in warm period of the year and low level in cold period. In previous studies stronger associations have been demonstrated for the warm period of the year between O_3_ exposure and CV and respiratory mortality [6, 11, 19, 28], while, we did not observe seasonal influence on our risk estimates.

Inflammation and oxidative stress are integral components in the pathogenic process of respiratory [29] but also of atherosclerotic heart disease [30]. Ozone has a high oxidative potential and has in animal studies been shown to adversely react with airway epithelium inducing smooth muscle hyperplasia and dysfunction, and contribute to subsequent bronchial hyperresponsiveness [31]. An experimental study in healthy young adults reported increased inflammatory markers and decreased lung function measures following exposure to O_3_ [32]. Other experimental studies have reported increase in levels of inflammatory markers such as pro-inflammatory cytokines and C-reactive protein after exposure to O_3_ [33–36]. Ozone exposure has also been associated with alterations in the autonomic nervous system [37–41] and changes in blood pressure in individuals with [42] and without [43] previous cardiovascular disease. In addition to possibly contributing to the risk of developing atherosclerotic heart disease, O_3_ exposure may be involved in the exacerbation of these disease processes in post-AMI patients.

This study has several limitations. Like most epidemiological studies, we did not have information on individual-specific O_3_ exposure, instead we used ambient concentrations from a single monitoring station as exposure estimates for the entire population. Moreover, we did not have data regarding the time spent indoors, activity pattern, and location of residents, which could introduce potential misclassification; however, this type of misclassification is likely non-differential and would bias towards the null. Although our dataset included possibility for multiple hospitalizations for several diseases we created mutually exclusive subpopulations for comparison purposes and could not take into account the full complexity of presence of multiple diseases. Our study was based exclusively on fatalities occurring during the study period and therefore precluded studying associations of non-fatal disease incidence. The major strength of our study is the full coverage of the mortality and previous hospitalization registries. These registries have been shown to have a high validity [10]. Although some level of inaccuracy is still expected in the registry data, it is not expected to vary with our exposure and thus the net result would be an underestimation of the observed estimates.

## Conclusion

Short-term O_3_ exposure was associated with CV and respiratory mortality. We found suggestive evidence of increased of susceptibility for CV mortality following short-term exposure to O_3_ in individuals previously hospitalized for AMI and observed an indication of higher risks in individuals with out-of-hospital CV death as their first presentation of disease. These results may support the consideration of increased risk in large susceptible subpopulations in health impact assessments of O_3_ exposure.

## Additional file

Additional file 1: **Table S1.** Two pollutant models. Percent change in cardiovascular and respiratory mortality associated with a 10 μg/m^3^ increase in 2-day and 7-day average air pollution concentrations, respectively in time series analyses. **Table S2.** Percent change in cardiovascular mortality associated with a 10 μg/m^3^ increase in 2-day average 8-h maximum O_3_ concentrations by season, age, and sex in case-crossover analyses. **Table S3.** Percent change in respiratory mortality associated with a 10 μg/m^3^ increase in 7-day average 8-h maximum O_3_ concentrations by season, age, and sex in case-crossover analyses. (DOCX 25 kb)

## Abbreviations

AMI: Acute myocardial infarction
CI: Confidence interval
COPD: Chronic obstructive pulmonary disease
CV: Cardiovascular
CVD: Cardiovascular disease
Df: Degrees of freedom
ICD-10: International Classification of Diseases, Tenth Revision
ICD-9: International Classification of Diseases, Ninth Revision
IQR: Interquartile Range
NO_2_: Nitrogen Dioxide
NO_x_: Nitrogen dioxides
O_3_: Ozone
PM_10_: Particles with aerodynamic diameter smaller than10 μm
PM_2.5_: Particles with aerodynamic diameter smaller than 2.5 μm
SD: Standard Deviation
