# Supplementary material for: Susceptibility to short-term ozone exposure and cardiovascular and respiratory mortality by previous hospitalizations
Source: Environ Health. 2018 Apr 13;17:37. doi: 10.1186/s12940-018-0384-z (PMC5899411; doi:10.1186/s12940-018-0384-z)
Supplement: Supplementary file 1 — Table S1. Two pollutant models. Percent change in cardiovascular and respiratory mortality associated with a 10 μg/m3 increase in 2-day and 7-day average air pollution concentrations, respectively in time series analyses. Table S2. Percent change in cardiovascular mortality associated with a 10 μg/m3 increase in 2-day average 8-h maximum O3 concentrations by season, age, and sex in case-crossover analyses. Table S3. Percent change in respiratory mortality associated with a 10 μg/m3 increase in 7-day average 8-h maximum O3 concentrations by season, age, and sex in case-crossover analyses. (DOCX 25 kb) [file 12940_2018_384_MOESM1_ESM.docx]

**Table S1.** Two pollutant models. Percent change in cardiovascular and respiratory mortality associated with a 10 μg/m^3^ increase in 2-day and 7-day average air pollution concentrations, respectively in time series analyses.

| Two pollutant models | Cardiovascular mortality (2-day) | | Respiratory mortality (7-day) | |
| --- | --- | --- | --- | --- |
|  | Percent change | 95 % CIs | Percent change | 95 % CIs |
| O_3_ | 0.9 | (0.2, 1.6) | 1.8 | (-0.4, 4.0) |
| PM_10_ | -1.3 | (-2.4, -0.2) | -0.9 | (-3.3, 1.8) |
|  |  |  |  |  |
| O_3_ | 1.3 | (0.3, 2.2) | 0.3 | (-2.7, 3.4) |
| PM_2.5_ | -1.2 | (-3.4, 1.0) | -6.2 | (-12.9, 1.0) |
|  |  |  |  |  |
| O_3_ | 0.9 | (0.2, 1.6) | 2.3 | (0.3, 4.3) |
| NO_x_ | 0.2 | (-0.2, 0.8) | -0.5 | (-2.2, 1.1) |
|  |  |  |  |  |
| O_3_ | 0.8 | (0.2, 1.5) | 2.4 | (0.5, 4.4) |
| NO_2_ | -0.2 | (-1.3, 1.0) | -0.8 | (-4.7, 3.2) |

**Table S2.** Percent change in cardiovascular mortality associated with a 10 μg/m3 increase in 2-day average 8-h maximum O3 concentrations by season, age, and sex in case-crossover analyses.

| Effect modifier | Percent change | 95 % CIs | p-value for interaction |
| --- | --- | --- | --- |
| Age |  |  |  |
| ≤ 82 years | 0.6 | (-0.2, 1.4) | 0.04 |
| > 82 years | 1.7 | (0.9, 2.4) |  |
| Sex |  |  |  |
| Men | 1.2 | (0.4, 2.1) |  |
| Women | 1.1 | (0.3, 1.9) | 0.74 |
|  |  |  |  |
| Season |  |  |  |
| Warm | 1.1 | (0.3, 2.0) | 0.9 |
| Cold | 1.2 | (0.4, 2.1) |  |
|  |  |  |  |

**Table S3.** Percent change in respiratory mortality associated with a 10 μg/m3 increase in 7-day average 8-h maximum O3 concentrations by season, age, and sex in case-crossover analyses.

| Effect modifier | Percent change | 95 % CIs | p-value for interaction |
| --- | --- | --- | --- |
| Age |  |  |  |
| ≤ 81 years | 4.0 | (1.3, 6.9) | 0.1 |
| > 81 years | 1.2 | (-1.2, 3.7) |  |
| Sex |  |  |  |
| Men | 2.1 | (-0.6, 4.8) | 0.6 |
| Women | 2.8 | (0.3, 5.5) |  |
| Season |  |  |  |
| Warm | 2.1 | (-0.3, 4.6) | 0.6 |
| Cold | 3.0 | (01, 5.9) |  |
